# The roles of circRNA–miRNA–mRNA networks in the development and treatment of osteoporosis

**DOI:** 10.3389/fendo.2022.945310

**Published:** 2022-08-05

**Authors:** Manqi Gao, Zhongkai Zhang, Jiabin Sun, Bo Li, Yuan Li

**Affiliations:** ^1^ Department of Pharmacy, Deqing People’s Hospital, Huzhou, China; ^2^ Department of Orthopedics, Shandong Provincial Hospital Affiliated to Shandong First Medical University, Jinan, China; ^3^ Department of Orthopedics, Sun Yat-Sen Memorial Hospital of Sun Yat-Sen University, Guangzhou, China; ^4^ Department of Pharmacology, School of Pharmaceutical Sciences, Cheeloo College of Medicine, Shandong University, Jinan, China; ^5^ Suzhou Research Institute, Shandong University, Suzhou, China

**Keywords:** circRNA, miRNA, osteoporosis, bone development, treatment

## Abstract

Osteoporosis is a systemic metabolic disease, mainly characterized by reduced bone mineral density and destruction of bone tissue microstructure. However, the molecular mechanisms of osteoporosis need further investigation and exploration. Increasing studies have reported that circular RNAs (circRNAs), a novel type of RNA molecule, play crucial roles in various physiological and pathological processes and bone-related diseases. Based on an in-depth understanding of their roles in bone development, we summarized the multiple regulatory roles and underlying mechanisms of circRNA–miRNA–mRNA networks in the treatment of osteoporosis, associated with bone marrow mesenchymal stem cells (BMSCs), osteoblasts, and osteoclasts. Deeper insights into the vital roles of circRNA–miRNA–mRNA networks can provide new directions and insights for developing novel diagnostic biomarkers and therapeutic targets in the treatment of osteoporosis.

## 1 Introduction

Osteoporosis (OP) is a common systemic metabolic disease, with high morbidity among the elderly especially in postmenopausal women (1). It is identified by the decreased bone toughness and altered bone microarchitecture, leading to an increase in skeletal fragility and fracture risk (2). Fracture is the most serious consequence of osteoporosis in the elderly, which easily leads to myeloid fracture, vertebral compression fracture, and other bone disorders. Osteoporosis has become one of the important causes of disability and death in the elderly due to its high incidence, high surgical risk, and poor prognosis (3, 4). With the aging of the population, osteoporosis has become a global health problem; it has been estimated that approximately 200 million individuals are affected by different levels of osteoporosis. The process of bone strength damage occurs silently and progressively, and there are no symptoms except fracture generally. Thus, the phenomenon not only has a socioeconomic impact and increases the healthcare cost related to osteoporosis, but also causes a heavy burden to patients with osteoporosis and their families (5, 6). However, there is no specific medicine to cure the disease in clinical practice, and the existing therapies can merely retard the process of osteoporosis and reduce the risk of fractures. Thus, it is imminently necessary to search for novel and effective therapeutics for osteoporosis.

Bone is a multifunctional, highly mineralized connective tissue acting as the center for the locomotory system and providing structural support for all internal organs. It is worth noting that bone is also a storeroom of calcium and phosphorus, which are prerequisite to maintain mineral homeostasis. Bone tissue metabolism is particularly active, and it endures constant remodeling during the whole life process. Bone remodeling is the process in which old bones are replaced by new bones, thereby maintaining bone mineral homeostasis and strength *via* a coupling between osteoblast-induced bone formation and osteoclast-mediated bone resorption (7–9). When the bones are affected by unfavorable factors, including aging, malnutrition, alcohol abuse, estrogen reduction, and connected adverse reactions to medications, the balance between bone formation and resorption will be broken, which is the main cause of generation osteoporosis (10, 11). However, because of a series of endogenous and exogenous factors included in bone metabolism, the particular molecular mechanism underlying the development of osteoporosis remains unclear (12–14).

**Figure 1:**
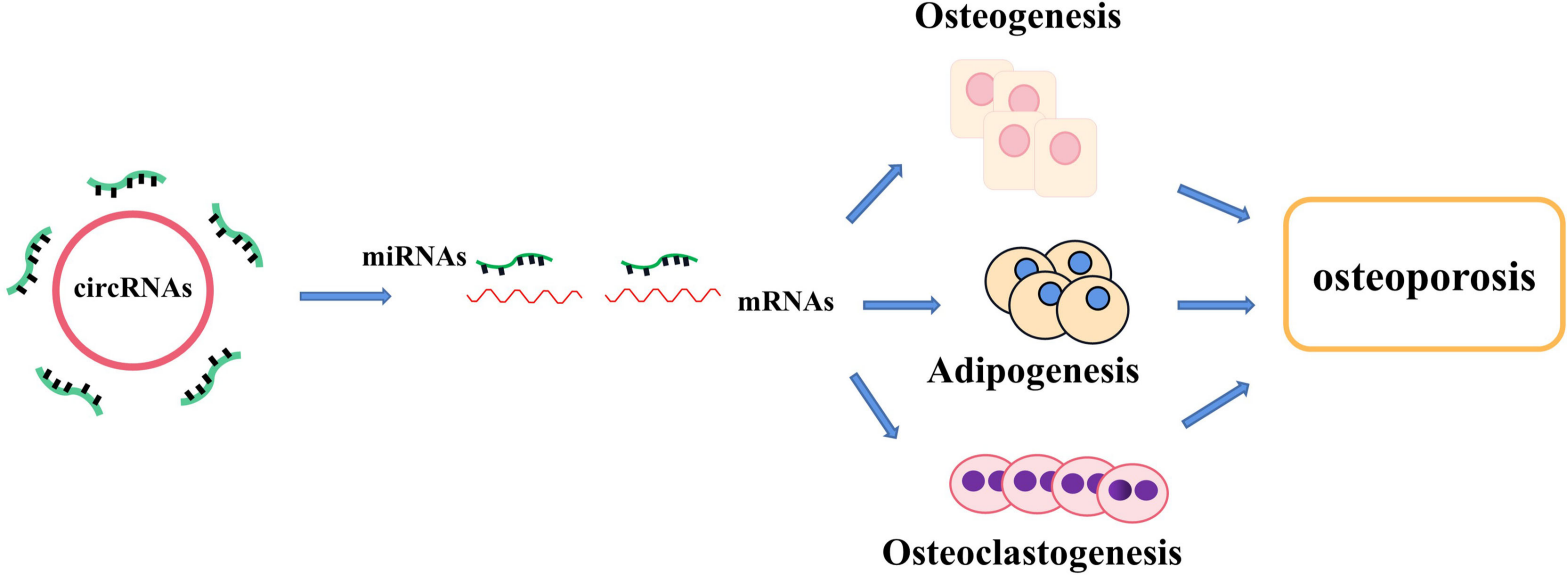
The regulatory roles of circRNA–miRNA–mRNA networks in bone development and bone homeostasis. CircRNA–miRNA–mRNA networks regulate the osteogenesis, adipogenesis, and osteoclastogenesis in several types of cells and provide a therapeutical approach to treating abnormal bone metabolism including osteoporosis.

Non-coding RNAs (ncRNAs), which are the novel focus point in most bioscience research, such as circular RNAs (circRNAs), microRNAs (miRNAs), and long ncRNAs (lncRNAs), have vital roles as regulators in various disease progressions, including osteoporosis (15, 16). CircRNAs, which widely exist in diverse organisms, emerge as a novel type of RNAs with a covalently closed-loop structure, implicated in the regulation of various biological activities, including cell growth, signaling, and multiple physiological and pathological responses (17). For example, circ_0000854, a novel circRNA, was detected to accelerate hepatocellular carcinoma (HCC) progression *via* the miR-1294/IRGQ axis, and silencing circ_0000854 suppresses cancer cell malignant behaviors, providing unique regulatory targets for HCC pathogenesis (18). Circ_SMG6 aggravated the resultant myocardial ischemia/reperfusion (I/R) injury, which might be related to the circ_SMG6-miR-138-5p-EGR1 network (19). This pathway can provide a novel therapeutic target to myocardial I/R injury (19). Meanwhile, emerging studies indicate that several circRNAs and miRNAs have been found to participate in the regulation of bone marrow mesenchymal stem cell (BMSC) differentiation and osteoporosis pathogenesis. It has been reported that circRNAs act as molecular sponges of miRNAs by competing for the rich miRNA-binding sites and interfering its expression with target messenger RNAs (mRNAs), which has been validated in various particular bone cellular activities (20). Moreover, the growing number of evidence proves that circRNA–miRNA–mRNA networks are a series of novel regulators in bone development (**Tables 1** and **Table 2**) (**Figure 1**). In this review, we comprehensively searched the PubMed database (https://pubmed.ncbi.nlm.nih.gov/) with the combined keywords “circRNA”, “miRNA”, and “osteoporosis”, and summarized the roles of circRNA–miRNA–mRNA networks in the development and treatment of osteoporosis.

**Table 1:** The auxo-action of the circRNA–miRNA–mRNA axis during bone formation.

CircRNAs	Target miRNAs	Target genes or pathways	Cell types	References
circRNA-0016624	miR-98	BMP2	hBMSCs	(21)
circRNA-0000020	miR-142-5p	BMP2	BMSCs	(22)
circRNA-0048211	miR-93-5p	BMP2	hBMSCs	(23)
circRNA-0007059	miR-378	BMP2	hBMSCs	(24)
circRNA-0006215	miR-942-5p	RUNX2/VEGF	BMSCs	(25)
mm9-circ-009056	miR-22-3p	BMP7	MC3T3-E1 cells	(26)
circRNA-Fgfr2	miR-133	BMP6	rDFCs	(27)
circRNA-AFF4	miR-135a-5p	FNDC5/Irisin and Smad1/5 pathway	BMSCs	(28)
circRNA-SIPA1L1	miR-617	Smad3	DPSCs	(29)
circRNA-RUNX2	has-miR-203	RUNX2	hBMSCs	(30)
circRNA-0011269	miR-122	RUNX2	hBMSCs	(31)
hsa-circ-0005752	miR-496	RUNX3	ADSCs	(32)
hsa-circ-33287	miR-214-3p	RUNX3	MSMSCs	(33)
hsa-circ-0026827	miR-188-3p	Beclin1 and the RUNX1	hDPSCs	(34)
circRNA-23525	miR-30a-3p	RUNX2	ADSCs	(35)
circRNA-0001795	miR-339-5p	YAP1	hBMSCs	(36)
circRNA-0024097	miR-376b-3p	YAP1 and Wnt/β-catenin pathway	BMSCs and MC3T3-E1	(37)
circRNA-Smg5	miR-194-5p	Fzd6 and β-catenin pathway	BMSCs	(38)
circRNA-124534	miR-496	β-Catenin Pathway	hDPSCs	(39)
circRNA-0006393	miR-145-5p	FOXO1	BMSCs	(40)
circRNA-FOXP1	miR-33a-5p	FOXP1	hASCs	(41)
circRNA-Rtn4	miR-146a	TNF-α	MC3T3-E1 cells	(42)
hsa-circ-0076906	miR-1305	OGN	hMSCs	(43)
circRNA-0062582	microRNA-145	CBFB	hBMSCs	(44)
hsa-circ-0006766	miR-4739	Notch2	hBMSCs	(45)
circRNA-vgll3	miR-326-5p	integrin α5	ADSCs	(46)
circRNA-AFF4	Mir-7223-5p	PIK3R1	MC3T3-E1 cells	(47)
hsa-circ-0008500	miR-1301-3p	PADI4	HEK and hFOB	(48)
hsa-circ-0074834	miR-942-5p	ZEB1 and VEGF	BMSCs	(49)
circRNA-SIPA1L1	miR-204-5p	ALPL	SCAPs	(50)
circRNA-DAB1	miR-1270 and miR-944	NOTCH/RBPJ pathway	hBMSCs	(51)

hBMSCs, human bone marrow mesenchymal stem cells; BMSCs, bone marrow mesenchymal stem cells; rDFCs, rat dental follicle cells; DPSCs, dental pulp stem cells; ADSCs, adipose-derived mesenchymal stem cells; MSMSCs, maxillary sinus membrane stem cells; hDPSCs, human dental pulp stem cells; BMMCs, bone marrow monocyte/macrophage cells; hASCs, human adipose-derived mesenchymal stem cells; hMSCs, human marrow mesenchymal stem cells; HEK, human embryonic kidney; hFOB, human osteoblast; SCAPs, stem cells from apical papillas.

**Table 2:** The inhibitory effects of the circRNA–miRNA–mRNA axis during bone formation.

CircRNAs	Target miRNAs	Target genes or pathways	Cell types	References
circRNA-POMT1 and circRNA-MCM3AP	miR-6881-3p	Smad6 and Chordin	hASCs	(52)
circRNA-HGF	miR-25-3p	Smad7	BMSCs	(53)
circRNA-0001052	miR-124-3p	Wnt4/β-catenin pathway	BMSCs	(54)
circRNA-CDR1as	miR-7-5p	WNT5B	BMSCs	(55)
circRNA-0006873	miR-142-5p	PTEN/Akt signaling pathway	hBMSCs	(56)
hsa-circ-0006859	miR-431-5p	ROCK1	hBMSCs	(57)
circRNA-009934	miR-5107	TRAF6	osteoclast	(58)
circRNA-25487	miR-134-3p	p21	BMSCs	(59)
hsa-circ-0001275	miR-377	CDKN1B	hFOB1.19 cells	(60)
circRNA-28313	miR-195a	CSF1	BMMCs	(61)
circRNA-0003865	miR-3653-3p	GAS1	BMSCs	(62)

hASCs, human adipose-derived mesenchymal stem cells; BMSCs, bone marrow mesenchymal stem cells; hBMSCs, human bone marrow mesenchymal stem cells; hFOB, human osteoblast; BMMCs, bone marrow monocyte/macrophage cells.

## 2 The roles of circRNA–miRNA–mRNA networks in the development of osteoporosis

### 2.1 Biogenesis and function of circRNAs

CircRNAs are a new type of non-coding RNAs with a closed continuous loop structure covalently *via* the concatenating of the 3’-poly(A) tails and 5’-end capping splice sites, which were first found in viroid in 1976 and widely distributed in nature (63). Compared with linear RNAs, circRNAs are more stable to exonuclease RNase Rand and highly conserved molecules (64). CircRNAs can be roughly classified into three categories: intronic circRNAs, exonic circRNAs, and exon–intron circRNAs, on the basis of the genome origin and their means of generation. Because of splicing errors, circRNAs were previously considered as nonfunctional by-products (64). In recent years, increasing circRNAs were investigated to function in a class of biological processes, such as cell proliferation, differentiation, and apoptosis (50, 61). According to a recent report, circFAM120B, as a tumor suppressor, hampers cell proliferation, metastasis, and invasion in esophageal squamous cell carcinoma (ESCC) (65). CircUbe3a could facilitate the cell proliferation, migration, and phenotypic transformation of cardiac fibroblasts (CFs) and then exacerbate myocardial fibrosis after acute myocardial infarction (66). Meanwhile, circRNAs have also been unexpectedly proven to take part in the regulation of bone microarchitecture, pathogenesis, and therapies of osteoporosis. Recently, a report has indicated that circ_0005564 significantly increased the mRNA levels of osteogenic differentiation markers, including RUNX2, OPN, and OCN, and then played energetic roles in osteoporosis (67). Nevertheless, the specific roles of various circRNAs in osteoporosis and the related underlying mechanisms need further exploration.

CircRNAs act as regulators in different essential physiological processes by regulating gene transcription, participating in translation, and acting as miRNA sponges. For instance, in a recent report, circStag1 was found to be related to osteoporosis (68). Overexpression of circStag1 could significantly promote the osteogenic capability in BMSCs, and researchers found that circStag1 directly interplays with an RNA-binding protein, named human antigen R (HuR), to mechanistically promote the regeneration of bone-related tissue (68).

### 2.2 Biogenesis and function of miRNAs

miRNAs are a kind of stem ring endogenous small non-coding RNA molecules, about 22 nucleotides, and present in all eukaryotic cells (69). miRNAs play a key role in the translation and expression of gene in organisms post-transcriptionally *via* combining the 3’-UTR region of mRNA and then silencing the expression of target genes (70). The expression of miRNAs exerts the characteristics of tissue specificity and growth process specificity, and they participate in cell proliferation, differentiation, apoptosis, and other development. When the content of some miRNAs changes abnormally, the corresponding diseases will be induced subsequently on account of the abnormal expression levels of target genes (71). It has been reported in the literature that the disorder of several miRNAs is closely related to the occurrence and development of many types of diseases (72, 73). For instance, in cancer, miRNAs can act not only as oncogenes but also as tumor suppressors. At the same time, miRNAs can also be used as markers in the process of cancers, providing new targets for cancer therapy (74). In recent years, the roles of miRNAs in osteoporosis have gradually been recognized. miRNAs can regulate bone metabolism by regulating osteogenic differentiation, osteoclast differentiation, and maturation. Therefore, the abnormal expression of miRNAs, related to bone metabolism, must be closely related to the occurrence of bone-related diseases, such as osteoporosis. According to the study, the level of miR-27a has been verified to be reduced in the disease process of osteoporosis and during adipogenic differentiation (75). Further research found that silencing of miR-27a could decrease bone formation *in vivo*. Therefore, in-depth study of these miRNAs will be helpful to understand the pathogenesis of bone replacement and osteoporosis and search the clinical diagnosis and treatment of osteoporosis.

### 2.3 The effects of circRNA–miRNA–mRNA networks as signaling pathway factors in bone development

#### 2.3.1 BMP signaling pathways

**Figure 2:**
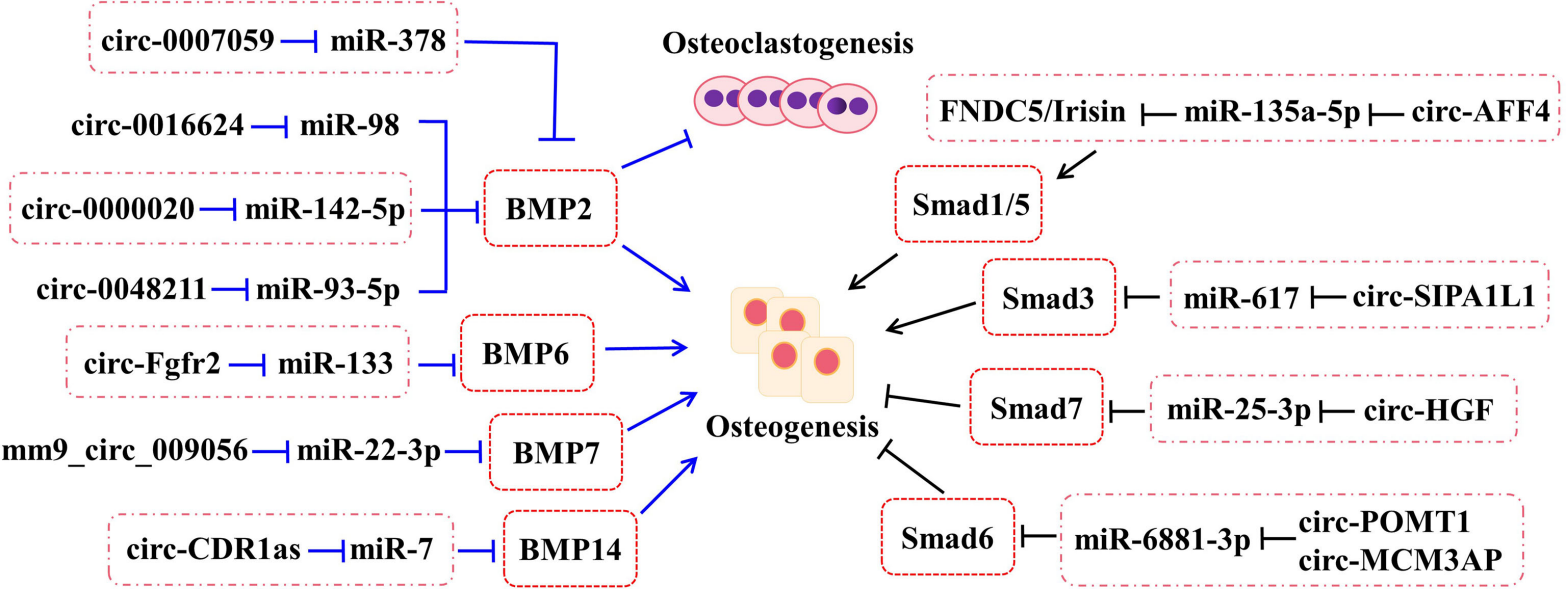
The roles of circRNA–miRNA–mRNA networks related to BMP and Smad pathways in bone development. The circRNA–miRNA–mRNA networks exert the potentials to regulate the bone development by BMP and Smad pathways.

Bone morphogenetic proteins (BMPs), one of the members of transforming growth factor-β (TGF-β) superfamily, undertake a pivotal role in regenerating osteogenic differentiation of osteoblast by Smads-related or non-Smads-related pathways. BMP receptors can receive information from BMP ligands, form a complex with co-Smad4 through phosphorylating R-Smad1/5/8, and affect the transcriptional expression of downstream target genes. According to the research, BMP2, BMP6, BMP7, and BMP14 (also named to as GDF5) have been affirmed to be correlated with bone homeostasis (**Figure 2**).

##### 2.3.1.1 BMP2

As a positive cytokine, bone morphogenetic protein-2 (BMP2) is involved in a variety of cellular activities, which is known to induce osteogenic differentiation and regulate bone development and fracture repair. For example, it is confirmed that circ-0000020 could promote osteogenic differentiation, retard the progress of osteoporosis, and upregulate the expression of BMP2 *via* sponging miR-142-5p as ceRNA (22). Inversely, the silence of circ-0000020 significantly decreased the expression of osteogenic markers, reduced the mineralization ability, and enhanced the apoptosis levels of BMSCs (22). Postmenopausal osteoporosis (PMO), one type of osteoporosis, caused by reduction of estrogen, dramatically reduces the quality of later life of postmenopausal women. According to the study, circ-0007059 was found by screened different circRNA expression levels in PMO patients *via* RNA-seq and bioinformatics analysis (24). Meanwhile, overexpression of circ-0007059 could attenuate osteoclastogenesis in hBMSCs *in vitro* (24). Further mechanism studies demonstrated that circ-0007059 directly targeted miR-378, which, in turn, targeted BMP2 (24). Hence, circ-0007059 was verified to function as a novel target in osteoclastogenesis *via* the miR-378/BMP2 signaling pathway (24). Furthermore, circRNA-0048211 could upregulate the expression of osteogenic genes during bone remodeling, including RUNX2, OPN, and OCN, and alleviate the progression of PMO through the circRNA-0048211/miRNA-93-5p/BMP2 regulatory network (23). The expression levels of circRNA-19142 and circRNA-5846 were observably upregulated in the BMP2-induced osteogenesis group compared with the control group (76). miR-7067-5p was confirmed to the co-targeted miRNAs of the two circRNAs by Venny analysis (76). Both circRNA-19142 and circRNA-5846 have been found to be involved in osteogenic activity through the circRNA-19142/cirRNAc-5846-miRNA-mRNA axis (76). Similarly, the circRNA-0016624/miR-98/BMP2 axis could prevent osteoporosis and offer a novel insight into therapeutic strategy (21).

##### 2.3.1.2 BMP6

CircRNA-Fgfr2 could sponge miR-133 and regulate the expression of bone morphogenetic protein-6 (BMP6) (27). For further validation, overexpression of circRNA-Fgfr2 in rDFCs restrained the expression of miR-133; on the contrary, the level of BMP6 was increased (27). Furthermore, during osteogenic induction, the positive regulation of the circRNA-Fgfr2/miR-133/BMP6 regulatory pathway has been verified (27).

##### 2.3.1.3 BMP7

Bone morphogenetic protein-7 (BMP7) belongs to the BMP family and possesses equivalent osteogenesis function. The calcitonin gene-related peptide (CGRP) could obviously promote the osteogenesis of MC3T3 cells and the expression of mm9_circ_009056 was significantly increased in the CGRP-induced cells (26). Furthermore, it could accelerate the expression levels of miR-22-3p following silencing mm9_circ_009056, but decrease the mRNA and protein levels of BMP7 (26). This phenomenon suggested that mm9_circ_009056 might act as a sponge for miR-22-3p to regulate the expression of BMP7 and then affect osteogenesis-related genes.

##### 2.3.1.4 GDF5

Growth differentiation factor (GDF) 5, also called bone morphogenetic protein-14 (BMP14), has been reported to participate in the process of osteogenic differentiation. The cerebellar degeneration-related protein 1 transcript (CDR1as), as a newly reported circRNA, has been discovered to be involved in the osteogenic differentiation of periodontal ligament stem cells (PDLSCs). By *in vivo* and *in vitro* assays, they found that circ-CDR1as plays the role of a miR-7 sponge, leading to upregulate the level of GDF5 and phosphorylate the Smad1/5/8 and p38 mitogen-activated protein kinases (p38 MAPK) mechanistically (77).

##### 2.3.1.5 Others

According to the latest studies, several circRNAs play a part in bone formation-related diseases through the Smad pathway (**Figure 2**). For example, circ_AFF4 sponges miR-135a-5p and modulates osteoblast differentiation by activating the Smad1/5 pathway *via* the miR-135a-5p/FNDC5/Irisin network in BMSCs (28). Both circPOMT1 and circMCM3AP have been demonstrated to crosstalk with hsa-miR-6881-3p. Moreover, hsa-miR-6881-3p possibly inhibits Smad6 to activate the BMP signaling pathway and influences osteogenesis of hASCs (52). At the same time, circRNA-SIPA1L1 accelerates osteogenesis in DPSCs *via* adsorbing miR-617 and further activating the Smad3 pathway (29). Moreover, circHGF, which was significantly upregulated in osteonecrosis of the femoral head (ONFH) sample group compared with the control group by circRNA microarray assay, restrained the cell proliferation and differentiation in BMSCs by acting on the miR-25-3p/Smad7 regulatory pathway (53).

#### 2.3.2 RUNX signaling pathways

##### 2.3.2.1 RUNX2

Runt-related transcription factor 2 (RUNX2), as a critical osteogenic marker gene, is broadly used to observe the osteogenic differentiation process. RUNX2 is involved in osteoblast differentiation through coordinating multiple signaling pathways, including BMPs, TGFs, Wnts, and hedgehogs. In the meantime, the upregulated level of RUNX2 can activate the expression of downstream Osterix, Col I, ALP, and other osteogenic genes. Yin et al. showed that the expression level of circRUNX2, derived from osteoporotic bone tissues, was downregulated (30). CircRUNX2 could be combined with miR-203 and compete for binding sites on miR-203 with RUNX2 (30). Overexpression of circRUNX2 could promote the osteogenic differentiation and restrain the progression of osteoporosis (30). A subsequent study has confirmed that circ-VANGL1, miR-217, and RUNX2 are correlated by the dual-luciferase reporter gene assay (78). At the same time, overexpression of circ-VANGL1 could lead to the increase of miR-217 and the decrease of RUNX2 and then the acceleration of osteogenic differentiation (78). Moreover, the expressions of bone formation-related genes were upregulated and ALP activity was increased by overexpressing circ-VANGL1 in hBMSCs (78). The reduced osteogenic ability is one of the pivotal causes of age-related osteoporosis in BMSCs. Overexpression of hsa_circ_0006215 could enhance the expression of the osteogenesis-related genes, and the condition in the hsa_circ_0006215 knockdown group was reversed (25). The results of luciferase reporter and RNA pull-down assays revealed the relationship between hsa_circ_0006215 and miR-942-5p and RUNX2 (25). The results also showed that hsa_circ_0006215 could regulate the RUNX2 and VEGF expression *via* targeting miR-942-5p and then promote the osteogenic differentiation (25). Similarly, the current studies show that circRNA-23525 promotes osteogenic differentiation by sponging miR-30a-3p to regulate RUNX2 expression in ADSCs (35). Meanwhile, circ_0011269 functions as a ceRNA binding to miR-122 and then regulates the expression of RUNX2 and accelerates osteoporosis progression (31).

##### 2.3.2.2 RUNX1 and RUNX3

Hsa_circ_0026827, which is increased typically during the osteoblast differentiation of human dental pulp stem cells (hDPSCs), was surprisingly found to promote heterotopic bone formation *in vivo*. The mechanism of action is *via* Beclin1 and the RUNX1 signal channels by binding to miR-188-3p, providing a novel thinking for osteoporosis research (34).

It has been proven that hsa_circ_0005752 could regulate osteogenic differentiation, as verified by alkaline phosphatase (ALP) and alizarin red S (ARS) staining assays (32). The hsa_circ_0005752/miR-496/MDM2 network plays a significant role in accelerating osteogenic differentiation. It is worth noting that RUNX3 could enhance the level of hsa_circ_0005752 and then promote osteogenic differentiation (32). On the contrary, knockdown of hsa_circ_0005752 partially antagonizes the function (32). In the meantime, hsa_circRNA_33287 may also affect the osteogenesis under the control of the ceRNA mechanism in maxillary sinus membrane stem cells (MSMSCs), which combines miR-214-3p and Runx3 by forming the hsa_circRNA_33287/miR-214-3p/Runx3 circuit (33). These pathways furnish novel mentalities for bone regeneration and therapeutics for bone disease treatment, such as osteoporosis.

#### 2.3.3 Wnts

**Figure 3:**
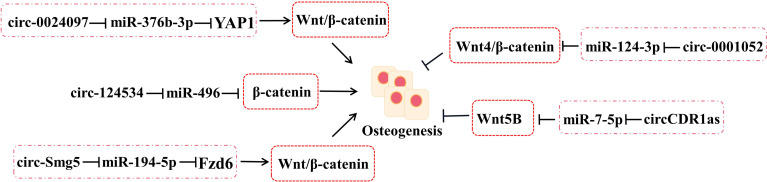
The roles of circRNA–miRNA–mRNA networks related to Wnt/β-catenin pathways in bone development. CircRNA–miRNA–mRNA networks participate in maintaining the bone development *via* Wnt/β-catenin pathways.

The interrelation between circRNA–miRNA–mRNA networks and Wnt signaling during osteogenic differentiation has been revealed (**Figure 3**). Wnt pathways are divided into the canonical and non-canonical Wnt/β-Catenin pathway.

Yes-associated protein 1 (YAP1), which belongs to YAP family, plays an important part in increasing bone mass and retarding bone microstructure degeneration in BMSCs and MC3T3-E1. Circ_0024097 could directly target miR-376b-3p and then attenuate the osteoporosis *via* both the circ_0024097/miR-376b-3p/YAP1 network and the Wnt/β-catenin pathway (37). Furthermore, WIF-1 acts as a rescue regulatory factor to change the positive effects of circ_0024097 overexpression on osteogenic differentiation, which is the inhibitor of the Wnt/β-catenin pathway (37). Another interesting circRNA for restraining osteoporosis is circRNA-124534, which promotes hDPSC bone regeneration *in vitro* and *in vivo* (39). Similarly, it works through the miR-496/β-catenin pathway to enhance the osteogenic differentiation (39). At the same time, circSmg5 could improve osteoblast differentiation *via* targeting the miR-194-5p/Fzd6 network to activate the Wnt/β-catenin pathway in BMSCs (38).

Several Wnts pertain to the non-canonical Wnt pathway, such as Wnt4, Wnt5a, and Wnt5b. According to the report, circRNA_0001052 is clarified to negatively regulate BMSC proliferation by binding to miR-124-3p as a sponger *via* the Wnt4/β-catenin pathway (54). It is worth mentioning that the low-level laser irradiation (LLLI) could reverse the process and provide a potential therapeutic to osteoporosis (54). The balance between osteogenic and adipogenic differentiation plays an important role in bone repair in BMSCs. miR-7-5p could link with CDR1as and WNT5B to modulate the osteogenic/adipogenic differentiation disorder, which may provide a novel direction for the molecular mechanisms of bone metabolism-related diseases (55).

## 3 The treatment of osteoporosis

### 3.1 Prevention

Osteoporosis is a type of chronic disease that is hardly cured at the present stage. Therefore, it is essential to prevent osteoporosis in advance. Calcium and vitamin D are essential components of skeletons and also the basic strategy for prevention and treatment of osteoporosis, especially beneficial for reducing the risk of fractures in the elderly. Simultaneously, vitamin D is significant in the treatment of anti-resorption and anabolic bone formation. However, when dietary calcium and vitamin D are insufficient to meet the demand, the balance is affected. It is widely agreed upon that food supplementation or the use of drug supplements is necessary to improve calcium and vitamin D levels and then prevent the development of osteoporosis (79, 80). It is widely established that the progress of bone loss may be significantly delayed by focusing on a diet of milk and dairy products, leading to a healthy lifestyle, and taking appropriate calcium and vitamin supplements (81, 82).

### 3.2 Routine drug therapy

According to the mechanism of function, anti-osteoporosis drugs can be divided into bone resorption inhibitors, bone formation promoters, dual-action regulators, and others. Bone resorption inhibitors are constituted by various drugs, including estrogens, selective estrogen receptor modulators (SERMs), calcitonin, and bisphosphonates. While parathyroid hormone (PTH) and analogues belong to bone formation promoters and strontium salt drugs play dual roles in regulating the new bone formation (**Table 3**).

**Table 3:** The routine drug therapy in osteoporosis.

Bone resorption inhibiter drugs				
Drug category	Typical drug	Function	Adverse reaction	References
ERT	Estrogen	Reducing bone resorption and suppressing the apoptosis of osteoblasts and osteocytes	The adverse effects of uterus, breast, and cardiovascular system increases	(83)
SERMs	Raloxifene	Reducing vertebral fracture risk	Hot flushes and venous thromboembolism	(84)
Calcitonin	Migaixi nasal spray	Increasing BMD and relieve pain	Pruritus, epistaxis, and arthralgia	(85)
NBP	Alendronate	Reducing the risk of vertebral and hip fractures	Gastrointestinal disturbance, osteonecrosis of the jaw, and atypical femoral fractures	(86)
RANKL inhibitor	Denosumab	Simple to use, and reducing bone turnover markers	Discontinuation can result in a rebound of curative effect and loss of BMD	(87)
Cathepsin K inhibitors	Odanacatib and ONO-5334	Suppression of bone resorption markers	Pycnodysostosis	(88)
**Bone formation promoter drugs**				
**Drug category**	**Typical drug**	**Function**	**Adverse reaction**	**References**
PTH1R	Teriparatide	Reducing both vertebral and non-vertebral fractures in postmenopausal patients	Persistent hypercalcemia, transient bone loss in clinic, and osteosarcoma caused by receiving high dose in rodents	(89)
PTHrP	Abaloparatide	Enhancing bone mass and lowers the risk of fracture	High cost and palpitations and heart rate increase	(90)
DKK-1 antibody	DKK-1 antibody	The Wnt-β-catenin signaling pathway physiological antagonists and increasing bone formation	Bone safety issues need further research	(91)
**Dual-action drugs**				
**Drug category**	**Typical drug**	**Function**	**Adverse reaction**	**References**
Strontium salt	Strontium ranelate	Intensify osteoblastogenesis while inhibiting osteoclastogenesis	The risk of myocardial infarction increases	(92)
Sclerostin antibodies	Romosozumab and blosozumab	Increasing bone formation and decreases bone resorption	Concern about the cardiovascular safety profile	(93)
GLP-1RAs	GLP-1RAs	Promoting bone formation and inhibiting bone resorption	The impact of fracture risk and osteoporosis needs to be further explored	(94)

ERT, estrogen replacement therapy; SERM, selective estrogen receptor modulator; NBP, nitrogen-containing bisphosphonate; RANKL, receptor activator of NF-κB ligand; PTH, parathyroid hormone; PTH1R, PTH-1 receptor; PTHrP, PTH-related protein; DKK-1, dickkopf-1; GLP-1RAs, Glucagon-like peptide-1 receptor agonists.

#### 3.2.1 Estrogens and SERMs

Estrogen is a steroid sex hormone produced by the endocrine system, which is involved in the physiological and pathological processes of bone tissue and plays an important role in the process of bone reconstruction. Estrogen reduces bone resorption *via* inhibiting the function of osteoclasts, and similarly selective estrogen receptor modulators are synthetic non-hormonal substances that play the same role in bone metabolism. The typical representative, raloxifene, can significantly increase the bone density and reduce the risk of fracture. However, the risk of breast cancer will increase because of long-term estrogen use. Therefore, long-term estrogen treatment is not recommended (85).

#### 3.2.2 Calcitonin

Calcitonin is a type of peptide hormone derived from thyroid cells and plays a vital role in the treatment of osteoporosis by directly inhibiting the differentiation and proliferation of osteoclasts. Then, calcitonin reduces the number of osteoclasts and destroys the dynamic balance of bone homeostasis. Calcitonin could also increase the bone density and relieve pain in osteoporosis. However, adverse reactions occur in several patients, and the effect of calcitonin on osteoclasts is transient, which exerts the obvious promotion in postmenopausal women (95).

#### 3.2.3 Strontium salts

Strontium salts exhibit multidirectional effects on bone tissue, not only promoting osteogenesis but also inhibiting bone resorption and contributing to reduced possibility of fracture. However, similar to major existing drugs, the disadvantage is obvious. The risk of myocardial infarction will dramatically increase after long-term administration in postmenopausal women (96). Strontium ranelate (SR) is a type of anti-osteoporosis drug with a multidirectional mechanism of action caused by strontium ions, which is similar to calcium in physical and chemical functions, and then plays a key role in regulating bone metabolism *via* the calcium-sensing receptor (CaSR) (92).

### 3.3 Novel drug therapies

In recent years, with more in-depth research on the pathogenesis of osteoporosis, a series of therapeutic targets has been developed, and new drugs have been gradually applied in clinical trials.

#### 3.3.1 PTH analogues

Essentially, PTH is a single-chain polypeptide secreted by the main cells of the parathyroid gland and joins in the regulative process of calcium homeostasis. Discontinuous action of PTH will stimulate bone formation and increase bone mass, but long-term action will lead to opposite effects, which are bone resorption enhancement and bone mass reduction (97). Recombinant human parathyroid hormone 1-34 (PTH1-34), which is also called teriparatide, is an active fragment of PTH. Teriparatide is the representative drug in osteoporosis and significantly reduces both vertebral and non-vertebral fractures in postmenopausal patients with osteoporosis (98).

#### 3.3.2 RANKL inhibitors

Denosumab, approved in 2010, competitively binds to receptor activator of NF-κB ligand (RANKL), thereby preventing merging with its receptor, RANK, and then inhibits osteoclast differentiation and activation, also reducing the osteoclastic activity. In clinical studies, it has been found that denosumab brings positive effects to higher spine BMD in the patient with postmenopausal osteoporosis. Dinoselmer is easy to use and a large number of clinical trials have proved that it can significantly reduce bone turnover markers (87, 99). However, discontinuation of denosumab can result in a rebound of the markers and even loss of accrued BMD.

#### 3.3.3 Cathepsin K Inhibitors

Cathepsin K is a member of the family of cysteine proteases, which has the highest expression and bone dissolution in osteoclasts. It decomposes bone tissue by degrading osseocolla and promotes the formation of cavities in bone, and then generates bone homeostasis disorder. Fortunately, two cathepsin K inhibitors, named odanacatib and ONO-5334, have been currently applied in the clinical treatment of osteoporosis. Such medicines will offer new therapeutic options for osteoporosis patients. Odanacatib is a reversibly selective, orally administered cathepsin K inhibitor. Odanacatib could reduce bone resorption and increase BMD and eventually achieve the goal of treating the disease *via* inhibiting the absorptive activity of osteoclasts, rather than decreasing their number (100). The other inhibitor, ONO-5334, is affirmed to exert the action of increasing BMD and bone strength parameters in the osteoporosis models of ovariectomized monkey and is associated with suppressing bone resorption markers in clinical settings (88). Hence, ONO-5334 was demonstrated as a late-model mode of function which could be used as a potential agent for the treatment of osteoporosis.

#### 3.3.4 Sclerostin antibodies

Sclerostin, a regulatory factor that affects bone remodeling, is secreted only by mature osteocytes and restrains osteogenesis by binding low-density lipoprotein receptor-related protein 5 (LRP5) and LRP6 receptors to inhibit the Wnt/β-catenin pathway (91). Sclerostin monoclonal antibodies, such as romosozumab and blosozumab, have been verified to inhibit sclerotin activity and reduce the sclerotin inhibition of Wnt signaling in multiple animal models. In the meantime, romosozumab could significantly increase the bone mass and reduce fracture risk by reducing bone resorption and promoting bone formation in comparison with alendronate at the lumbar spine (101, 102). Sclerostin antibody shows promise in the treatment of established osteoporosis, although there are some existing problems that need to be solved, including optimal duration and order of administration (103).

#### 3.3.5 CircRNA–miRNA–mRNA networks

As the research progressed, the role of circRNA–miRNA–mRNA networks in regulating bone metabolism balance and diagnosing bone-related diseases, and even in providing new ways of treatment to osteoporosis, was gradually proposed. For example, both the circ_0001795/miR-339-5p/YAP1 axis and the hsa_circ_0006766/miR-4739/Notch2 axis could attenuate osteoporosis progression and could be used as potential candidate therapeutic targets of bone regenerative medicine (36, 45). On the contrary, the circ_0006873-miR-142-5p-PTEN/Akt signaling pathway and the hsa_circ_0006859/miR-431-5p/ROCK1 signaling pathway could reverse the positive effects of osteogenic differentiation and promote the development of osteoporosis, which may provide treatment approaches for osteoporosis (56, 57). Overall, these results are helpful in exploring the pathogenesis of osteoporosis and in providing new strategies for its treatment.

## 4 Conclusions and perspectives

Above all, osteoporosis is a kind of complex multiple-factor chronic disease that needs further research to solve the problem of pathogenesis. Along with the advancement of osteoblast and osteoclast biology research, researchers have found a variety of regulatory transcription factors and signal pathways in the development of osteoporosis, including circRNA–miRNA–mRNA networks. The circRNA-associated ceRNA networks are closely related to the pathogenesis of osteoporosis by regulating osteoporosis-related key genes. Based on these findings on circRNA–miRNA–mRNA networks in osteoporosis, subsequent studies are needed to verify the application of circRNA–miRNA–mRNA networks in the prevention and treatment for osteoporosis. Targeting key circRNAs or miRNAs, identified as potentially crucial mechanisms underlying osteoporosis, might be a novel therapeutic method for osteoporosis.

Plenty of evidence suggest that these networks play significant roles in modulating the osteogenic/adipogenic differentiation in numerous types of cells and then dedicate to the osteoporosis. These findings are dramatically helpful to improve the current understanding of circRNA–miRNA–mRNA networks’ function in osteogenesis. In addition, existing drugs have obvious adverse reactions or are not suitable for long-term use. Therefore, the adverse reactions limit the longer and widespread use of these drugs. With the emergence of more in-depth studies, new target drugs are gradually proposed, which might be more effective in maintaining the dynamic balance in bone reconstruction, reducing the patient’s pain and improving the quality of life. Meanwhile, the corresponding therapeutics of circRNA–miRNA–mRNA networks are hoped to be applied in clinic and might be novel therapeutic targets in osteoporotic patients. As we mentioned, overexpression or knockdown of key circRNA/miRNAs might be a novel therapeutic strategy for osteoporosis. These important circRNA/miRNAs may be applied as a potential target to develop promising anti-osteoporotic drugs. However, the functions of more potential circRNA–miRNA–mRNA networks in the development of osteoporosis should be verified. A deep understanding of the osteoporosis-specific dysregulated circRNA–miRNA–mRNA network-mediated gene regulation in the development of osteoporosis is necessary to lay a firm foundation to develop promising therapeutic targets for osteoporosis.

## Author contributions

MG conceived the review paper with the guidance of YL and BL. MG wrote the original draft. MG, ZZ, JS, BL, and YL reviewed and edited the paper. All authors have read and agreed to the published version of the manuscript. All authors contributed to the article and approved the submitted version.

## Funding

This work was supported by the Postdoctoral Science Foundation of China (2021M691956), the Natural Science Foundation of Shandong Province (ZR2021QH087), the Medicine and Health Science and Technology Development Plan Project of Shandong Province (202002040581), the Natural Science Foundation of Jiangsu Province (BK20210110), and the Science and Technology Development Project of Suzhou (SYG202119).

## Conflict of interest

The authors declare that the research was conducted in the absence of any commercial or financial relationships that could be construed as a potential conflict of interest.

## Publisher’s note

All claims expressed in this article are solely those of the authors and do not necessarily represent those of their affiliated organizations, or those of the publisher, the editors and the reviewers. Any product that may be evaluated in this article, or claim that may be made by its manufacturer, is not guaranteed or endorsed by the publisher.
